# How does baseline anthropometry affect anthropometric outcomes in children receiving treatment for severe acute malnutrition? A secondary analysis of a randomized controlled trial

**DOI:** 10.1111/mcn.13329

**Published:** 2022-02-14

**Authors:** Clarisse Dah, Millogo Ourohire, Ali Sié, Moussa Ouédraogo, Mamadou Bountogo, Valentin Boudo, Elodie Lebas, Fanice Nyatigo, Benjamin F. Arnold, Kieran S. O'Brien, Catherine E. Oldenburg

**Affiliations:** ^1^ Centre de Recherche en Santé de Nouna Nouna Burkina Faso; ^2^ Francis I Proctor Foundation University of California San Francisco California USA; ^3^ Department of Ophthalmology University of California San Francisco California USA; ^4^ Department of Epidemiology & Biostatistics University of California San Francisco California USA

**Keywords:** mid‐upper arm circumference, screening, severe acute malnutrition, wasting, weight‐for‐height *Z*‐score

## Abstract

Mid‐upper arm circumference (MUAC) < 11.5 cm and weight‐for‐height *Z*‐score (WHZ) < −3 are used for screening for severe acute malnutrition (SAM). Underweight and concurrent wasting and stunting may better target those at the highest risk of mortality. We compared anthropometric outcomes in children enrolled in a trial of antibiotics for SAM based on categories of baseline anthropometry, including indicators for programme admission (WHZ < −3, MUAC < 11.5) and alternative indicators (weight‐for‐age *Z*‐score [WAZ] < −3, concurrent wasting and stunting [WHZ < −3 and height‐for‐age *Z*‐score < −3]). Participants were followed weekly until nutritional recovery and at 8 weeks. We evaluated changes in weight gain (g/kg/day), MUAC, and WHZ in children admitted by admissions criteria (MUAC only, WHZ only, or MUAC and WHZ) and by underweight or concurrent wasting and stunting. Of 301 admitted children, 100 (33%) were admitted based on MUAC only, 41 (14%) WHZ only, and 160 (53%) both MUAC and WHZ, 210 (68%) were underweight and 67 (22%) were concurrently wasted/stunted. Low MUAC and low WHZ children had the lowest probability of nutritional recovery (17% vs. 50% for MUAC‐only and 34% for WHZ‐only). There was no difference in weight gain velocity or WHZ by admissions criteria (WHZ and/or MUAC). Underweight and concurrently wasted/stunted children had lower MUAC and WHZ at 8 weeks compared with those who were not underweight or concurrently wasted and stunted. Children with both low MUAC and low WHZ had the worst outcomes. Relying on MUAC alone may miss children who have poor outcomes. Other indicators, such as WAZ, may be useful for identifying vulnerable children.

## Key messages

- Mid‐upper arm circumference (MUAC) and weight‐for‐height Z‐score (WHZ) are currently used for severe acute malnutrition (SAM) screening, but alternative indicators such as weight‐for‐age Z‐score (WAZ) may be additional options.
- Children admitted to the nutritional program based on both low MUAC and low WHZ had the worst outcomes, including the lowest probability of nutritional recovery and the worst anthropometric outcomes.
- While MUAC identified most children with SAM, WHZ may provide additional information about poor outcomes beyond MUAC alone.
- WAZ may be a useful alternative indicator to identify children who would benefit from a therapeutic feeding program without requiring height measurement.

## Graphical abstract

Weight‐for‐height *Z*‐score (WHZ) and mid‐upper arm circumference (MUAC) are used for screening for severe acute malnutrition, but alternative indicators such as weight‐for‐age *Z*‐score (WAZ) may identify children at high risk of mortality. We evaluated anthropometric outcomes in children with severe acute malnutrition by baseline anthropometric status. Children with both low WHZ and MUAC had the worst outcomes, and low WAZ additionally identified children with poor outcomes

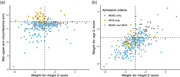

## 1 INTRODUCTION

Admission to outpatient nutritional programmes for uncomplicated severe acute malnutrition (SAM) is typically based on either a mid‐upper arm circumference (MUAC) of <11.5 cm and/or a weight‐for‐height *Z*‐score (WHZ) < −3 SD below 2006 World Health Organization standards (WHO Multicentre Growth Reference Study Group, 2006; World Health Organization, UNICEF, 2010). Recently, calls have been made to prioritize MUAC for screening and admission for outpatient SAM treatment (Briend et al., 2016). MUAC has been shown to be a stronger predictor of mortality than WHZ in some community‐based settings (Gao et al., 2020). MUAC measurement is simple and may facilitate community‐based screening and referral for treatment (Alé et al., 2016). However, MUAC and WHZ have been shown to identify different subgroups of children, and reliance solely on MUAC for diagnosis of SAM may miss a substantial proportion of children who would benefit from SAM treatment (Grellety et al., 2015; Schwinger et al., 2019). For example, one analysis of outcomes of children with SAM in South Sudan found that MUAC‐only screening would have missed approximately one‐third of children who ultimately died of SAM (Grellety et al., 2015). Markers of risk in children with SAM have also been shown to be increased in children admitted based on WHZ or WHZ and MUAC compared with MUAC only, including biomarkers such as leptin and serum ferritin and clinical markers such as dehydration (Dailey‐Chwalibóg et al., 2021). These studies suggest that although WHZ is functionally more challenging to measure than MUAC, it should continue to be included as a diagnostic criterion for SAM.

Currently, there is no gold standard for the diagnosis of SAM. Both MUAC and WHZ are imperfect measures of SAM and do not provide a definitive diagnosis (Kerac et al., 2020). These measures are meant to identify groups of children who are at the highest risk of mortality and morbidity and thus would benefit the most from treatment. Criteria for identifying children with SAM have changed over time, and alternative cut‐offs of currently used measures or alternative measures such as weight‐for‐age *Z*‐scores (WAZ) or concurrent wasting and stunting may be shown to better identify high‐risk children. Other clinical or nutritional factors, such as breastfeeding, may also significantly modulate the risk of poor outcome regardless of admission criterion.

Response to treatment for outpatient SAM may be different for children admitted based on different criteria given that these criteria identify different subpopulations of children. We utilized data from a randomized controlled trial of azithromycin compared with amoxicillin for outpatient management of SAM (O'Brien, Sié, Dah, Ourohire, Arzika, et al., 2021) to evaluate response to treatment in children admitted to outpatient nutritional programmes based on low MUAC, low WHZ, or low MUAC and WHZ. We further evaluated outcomes in children with low WAZ and height‐for‐age *Z*‐score (HAZ) at enrolment, as these markers may be useful in identifying the highest‐risk children who may benefit from admission to a nutritional programme.

## 2 METHODS

### 2.1 Study setting

Complete methods for the trial have been previously reported (O'Brien, Sié, Dah, Ourohire, Arzika, et al., 2021; O'Brien, Sie, Dah, Ourohire, Ouédraogo, et al., 2021) Participants were recruited via outpatient nutritional programmes at six primary health care facilities in Boromo District, Burkina Faso from June through October 2020. These facilities provide routine outpatient care for children with SAM that includes a package of interventions including ready‐to‐use therapeutic food (RUTF), antibiotics, antiparasitics, screening and treatment for malaria, and any vaccinations that the child is missing. The nutritional programmes typically offer screening and treatment services for SAM once per week and during curative consultations. In the catchment area for each primary health care facility, community health workers screen children for SAM using standard MUAC tapes and refer children with MUAC < 11.5 cm to the nutritional programme. Community‐based screening relies solely on MUAC‐based screening and community health workers go door‐to‐door to screen for acute malnutrition. Community health workers did not screen for SAM using WHZ. The study area has highly seasonal rainfall, with the rainy season lasting approximately July through October, which corresponds with the annual high malaria transmission season and the high food insecurity season, similar to much of the Sahel (Burki, 2013). Seasonal malaria chemoprevention is distributed monthly to all children aged 3 –59 months in the study area from July through October.

The study was reviewed and approved by the Comité Institutionnel d'Ethique in Nouna and the Institutional Review Board at the University of California, San Francisco. Written informed consent was obtained from the caregivers of all included participants.

### 2.2 Participants

Participants were eligible for the parent trial if they had a MUAC < 11.5 cm and/or WHZ < −3 at the time of screening, were between 6 and 59 months of age, had not been admitted to a nutritional programme for SAM in the previous 14 days, had no antibiotic use in the past 7 days, had no clinical complications requiring inpatient or antibiotic treatment, had sufficient appetite according to feeding test with RUTF, had no reported allergy to macrolides or azalides, had no congenital abnormality or chronic illness that would predictably lead to growth faltering and were planning to stay in the study area for the full 8‐week study period. Study staff recorded on the study's mobile data application whether participants were enrolled in the study based on MUAC‐ and/or WHZ‐based screening for admission to the nutritional programme, but specific values were not recorded. The admission criterion used for the nutritional programme was used to define exposure categories in the present analysis, and thus occasionally differs from how these children were categorized based on the trial's baseline anthropometric assessment, which occurred after screening and enrolment.

### 2.3 Anthropometric measurements

Anthropometric measurements were taken at each study visit. Participants were weighed (Seca 874dr scale; Seca) and had height (ShorrBoard Infant/Child Measuring Board; Weight and Measure, LLC) and MUAC (Shorr Child MUAC tape; Weight and Measure, LLC) in triplicate and the median was used for analysis. Weight gain was calculated in g/kg/day since baseline (primary outcome) and also as period‐specific weight gain, for example, baseline to Week 1, Weeks 1–2, Weeks 2–4 and Weeks 4–8. Weight and height measurements were used to calculate WHZ, WAZ and HAZ based on 2006 World Health Organization standards (WHO Multicentre Growth Reference Study Group, 2006).

### 2.4 Nutritional recovery, mortality, and morbidity

Vital status was assessed at each study visit, and children were marked as alive, dead, or vital status unknown in the study's mobile data application. Nutritional recovery was defined per Burkinabè guidelines as WHZ ≥ −2 and/or MUAC ≥ 12.5 cm on two consecutive visits, based on the criterion used for enrolment. A rapid diagnostic test (RDT) for malaria was performed on all children at 8 weeks after enrolment (Sié, Dah, et al., 2021). At each study time point, caregivers were interviewed to assess if their child had experienced any fever or diarrhoea since the last study visit. We categorized any report of fever or diarrhoea during the 8‐week study visit as the child having experienced fever or diarrhoea.

### 2.5 Statistical analysis

Anthropometric outcomes were summarized descriptively with means, SDs, and 95% confidence intervals (CIs) at baseline and Weeks 1, 2, 4, and 8. To assess if anthropometric outcomes were significantly different at 8 weeks, we used unadjusted and adjusted linear regression models for the admission criterion for the nutritional programme predicting each outcome (weight gain velocity in g/kg/day, MUAC, WHZ, WAZ and HAZ). Models were adjusted for the child's age, sex and the baseline measure for the outcome, with the exception of g/kg/day, which includes baseline weight in its calculation. To assess if there was a differential change in each anthropometric outcome over time, we used repeated‐measures models with an interaction term for the admissions criterion by study week adjusting for the child's age and sex. We then used unadjusted and adjusted logistic regression models to assess odds of achieving nutritional recovery by admissions criterion, with models adjusted for the child's age, sex and baseline MUAC and WHZ. Analyses pooled data from both trial arms and we did not adjust for the randomized treatment assignment because randomization and treatment happened after admission to the nutritional programme and adjustment for a post‐exposure variable may induce selection bias. There was no evidence of a difference in anthropometric outcomes by randomization arm in the trial's original analysis (O'Brien, Sie, Dah, Ourohire, Ouédraogo, et al., 2021).

We then evaluated outcomes described above among children who were severely underweight (defined as WAZ < −3) at baseline and those who were concurrently severely wasted and stunted (defined as WHZ < −3 and HAZ < −3) at baseline. Models were identical to those described using the admission criteria to the trial. Models for underweight and concurrent wasting and stunting were run separately, as their categories are not mutually exclusive. All analyses were conducted in Stata 15.1 (StataCorp).

## 3 RESULTS

**Figure 1:**
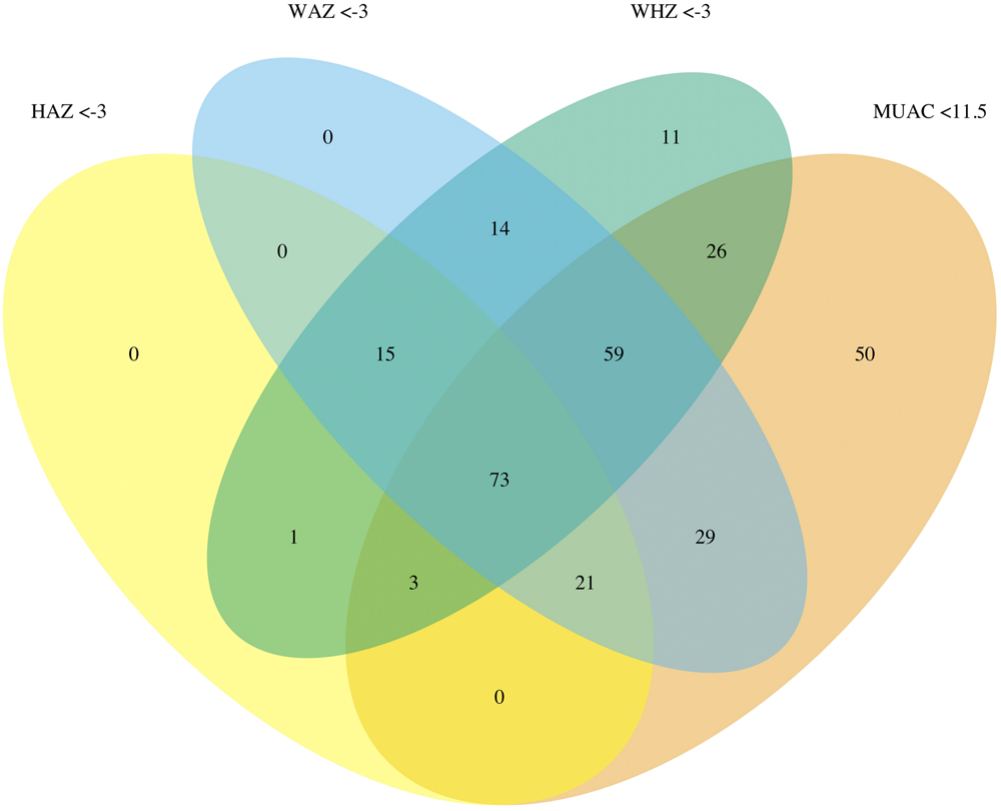
Venn diagram depicting the number of children enrolled in categories defined by criteria used for admission to the outpatient nutritional programme (weight‐for‐height *Z*‐score [WHZ] < −3, green, and/or mid‐upper arm circumference [MUAC] < 11.5 cm, brown) and weight‐for‐height *Z*‐score (WAZ) < −3 (blue) and height‐for‐age *Z*‐score (HAZ) < −3 (yellow) at enrolment

**Figure 2:**
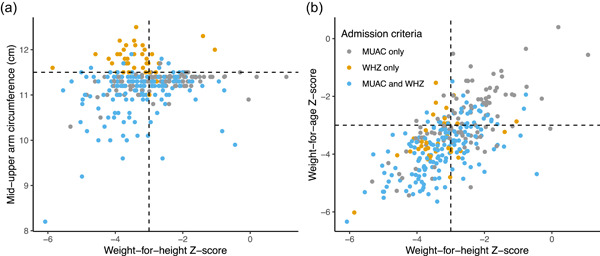
Scatter plots of enrolment weight‐for‐height *Z*‐score (WHZ) by mid‐upper arm circumference (MUAC; a) and WHZ by weight‐for‐age *Z*‐score (WAZ; b). Values are from the baseline anthropometric assessment, not admissions criteria. Points are values for individual children and are coloured by enrolment criterion met to enter the nutritional programme. Grey dots indicate the child met the criteria based on MUAC only, yellow indicates WHZ only and blue indicates MUAC and WHZ

Of 301 children enrolled in the trial, 100 were enrolled based on MUAC < 11.5 cm, 41 WHZ < −3, and 160 MUAC < 11.5 and WHZ < −3 (Figure 1 and Figure S1). Of the 301 children, 88 (29%) recovered by 8 weeks, 198 (66%) did not recover by 8 weeks, 12 (4%) were lost to follow‐up, and 3 (1%) died (Figure S1). Children enrolled with low MUAC only were a median 13 months old (interquartile range [IQR], 9.4–22.5 months) and 64% female, those with low WHZ only were median 19 months (IQR, 15–23 months) and 42.5% were female and those with both low MUAC and low WHZ were median 14 months (IQR, 10–22 months) and 55% were female (Table 1). As expected, baseline anthropometric indicators were low for all subgroups of children. At baseline, HAZ was lower for children enrolled with low MUAC and WHZ compared with those meeting only one of the criteria (WHZ and MUAC: mean HAZ −2.9; MUAC only: mean HAZ −1.8; WHZ only: mean HAZ −2.2; Table 1). Baseline WAZ was lower for those enrolled based on low WHZ and MUAC (WAZ −3.8) and WHZ only (WAZ −3.5) than MUAC only (WAZ −2.8; Table 1). Figure 2 shows scatter plots of baseline WHZ versus MUAC (Figure 2a) and WAZ (Figure 2b) as measured at the baseline assessment of the trial by admission criteria per programme staff. At baseline, among the 41 children who were enrolled based on WHZ only, 30 (73%) had MUAC < 12.0 cm and 39 (95%) had a MUAC measurement <12.5 cm. Of the two children admitted based on low WHZ without MUAC < 12.5 cm, one MUAC measurement was missing at baseline and the other had a MUAC of 12.5 cm.

**Table 1:** Baseline characteristics by admission criterion to an outpatient treatment programme for uncomplicated severe acute malnutrition (*N* = 301)

	MUAC only	WHZ only	MUAC and WHZ
*N*	100	41	160
Child's age, months, median (IQR)	13 (9.5–22.5)	19 (15–23)	14 (10–22)
Female sex, *N* (%)	64 (64)	17 (42.5)	88 (55)
Child's dietary diversity^a^, median (IQR)	7 (4.5–8.5)	6 (5–7)	6 (4–8)
Breastfeeding, *N* (%)	71 (71)	27 (66)	114 (71)
Household sanitation			
None/bush	22 (22%)	8 (20%)	15 (9%)
Unimproved latrine	32 (32%)	6 (15%)	43 (27%)
Improved latrine	45 (45%)	26 (65%)	77 (48%)
Flush toilet	1 (1%)	0 (0%)	25 (16%)
MUAC (cm), mean (SD)	11.3 (0.2)	11.8 (0.3)	11.1 (0.5)
WHZ, mean (SD)	−2.6 (1.1)	−3.4 (0.8)	−3.3 (0.9)
WAZ, mean (SD)	−2.8 (1.1)	−3.5 (0.8)	−3.8 (0.9)
HAZ, mean (SD)	−1.8 (1.5)	−2.2 (1.5)	−2.9 (1.4)
Mother's age (years), median (IQR)	24 (21–28)	25.5 (22–28)	26 (21–31)
Mother is literate	21 (21%)	5 (12.5%)	28 (17.5%)

Abbreviations: HAZ, height‐for‐age *Z*‐score; IQR, interquartile range; MUAC, mid‐upper arm circumference; WAZ, weight‐for‐age *Z*‐score; WHZ, weight‐for‐height *Z*‐score; SD, standard deviation.

^a^ Composite variable assessing whether the child ate foods from the following 11 food groups in the last 7 days: grains, orange vegetables, green leafy vegetables, mangoes, other fruits, other vegetables, animal protein, eggs, nuts, dairy or fat.

Among all enrolled children, 210 (70%) were underweight (WAZ < −3) and 67 (22%) were concurrently stunted (HAZ < −3) and wasted (WHZ < −3). There was substantial overlap in low WAZ and HAZ by admissions criteria based on WHZ and MUAC (Figure 1 and Figure S2). Children who were underweight and were stunted and wasted at baseline had substantial anthropometric deficits at baseline (Table S1). Among children admitted based on low WHZ only, 29 (71%) had WAZ < −3 and 38 (93%) had WAZ < −2.

**Figure 3:**
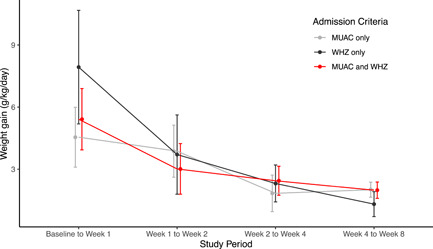
Weight gain in g/kg/day over time by criteria used for admission to the outpatient nutritional programme. Children admitted based on mid‐upper arm circumference (MUAC) < 11.5 cm only are in grey, those with weight‐for‐height *Z*‐score (WHZ) < −3 in black and those with both MUAC < 11.5 cm and WHZ < −3 in red. Points represent means for the study time point and bars represent 95% confidence intervals

**Figure 4:**
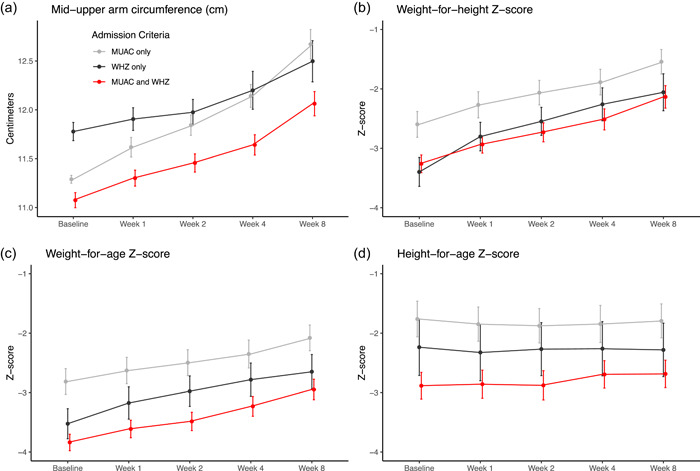
Mid‐upper arm circumference (MUAC; a), weight‐for‐height *Z*‐score (WHZ; b), weight‐for‐age *Z*‐score (WAZ; c), and height‐for‐age *Z*‐score (HAZ; d) over time by criteria used for admission to the outpatient nutritional programme. Children admitted based on mid‐upper arm circumference (MUAC) < 11.5 cm only are in grey, those with weight‐for‐height *Z*‐score (WHZ) < −3 in black, and those with both MUAC < 11.5 cm and WHZ < −3 in red. Points represent means for the study time point and bars represent 95% confidence intervals

There was no evidence of a difference in weight gain velocity by admission criteria over time (Figure 3; *p* for interaction = 0.15) or at 8 weeks (Table 2). MUAC improved more rapidly over time in children admitted based on MUAC only compared with both the WHZ‐only cohort and the MUAC and WHZ cohort (Figure 4a; *p* for interaction <0.001; *p* for main effect of time < 0.001). MUAC at 8 weeks was significantly lower in models adjusting for baseline MUAC in the WHZ only and MUAC and WHZ cohorts compared with MUAC only, reflecting that the children admitted based on MUAC only had more rapid changes in MUAC over time compared with the other cohorts (Table 2).

**Table 2:** Associations between admissions criteria and anthropometric outcomes at 8 weeks

	*N*	Mean (SD)	Unadjusted difference (95% CI)	Adjusted difference (95% CI)^a^
Weight gain (g/kg/day)				
MUAC only	96	2.5 (1.6)	Ref.	Ref.
WHZ only	37	2.7 (1.6)	0.2 (−0.5 to 0.9)	0.3 (−0.4 to 1.1)
WHZ and MUAC	148	2.6 (2.1)	0.1 (−0.4 to 0.6)	0.1 (−0.4 to 0.6)
MUAC (cm)				
MUAC only	96	12.7 (0.7)	Ref.	Ref.
WHZ only	37	12.5 (0.6)	−0.2 (−0.5 to 0.1)	−0.5 (−0.8 to −0.2)
WHZ and MUAC	148	12.1 (0.8)	−0.6 (−0.8 to −0.4)	−0.5 (−0.7 to −0.3)
WHZ				
MUAC only	96	−1.5 (1.0)	Ref.	Ref.
WHZ only	37	−2.1 (0.9)	−0.5 (−0.9 to −0.1)	−0.5 (−0.4 to 0.3)
WHZ and MUAC	148	−2.1 (1.2)	−0.6 (−0.9 to −0.3)	−0.2 (−0.5 to 0.02)
WAZ				
MUAC only	96	−2.1 (1.1)	Ref.	Ref.
WHZ only	37	−2.7 (0.9)	−0.6 (−1.0 to −0.2)	−0.01 (−0.3 to 0.3)
WHZ and MUAC	148	−2.9 (1.1)	−0.9 (−1.1 to −0.6)	−0.1 (−0.4 to 0.1)
HAZ				
MUAC only	96	−1.8 (1.4)	Ref.	Ref.
WHZ only	37	−2.3 (1.3)	−0.5 (−1.0 to 0.05)	−0.1 (−0.4 to 0.2)
WHZ and MUAC	148	−2.7 (1.4)	−0.9 (−1.3 to −0.5)	−0.1 (−0.3 to 0.1)

Abbreviations: CI, confidence interval; HAZ, height‐for‐age *Z*‐score; MUAC, mid‐upper arm circumference; SD, standard deviation; WAZ, weight‐for‐age *Z*‐score; WHZ, weight‐for‐height *Z*‐score.

^a^ Adjusted for child's age, sex and baseline value for the corresponding outcome (except for weight gain in g/kg/day as baseline values are incorporated into the outcome measure).

Although all three admissions criteria groups saw improvements in WHZ, there was no difference in change in WHZ over time by admission criteria (Figure 4b; *p* for interaction = 0.17; *p* for main effect of time < 0.001) and WHZ stayed consistently lower for children admitted based on WHZ only and MUAC and WHZ. Similarly, WAZ increased over time for all three groups at a similar rate (Figure 4c; *p* for interaction = 0.24; *p* for main effect of time < 0.001). HAZ did not change over time in any of the three admissions groups (Figure 4d; *p* for interaction = 0.22; *p* for main effect of time = 0.65). Children who were admitted based on both MUAC and WHZ had consistently lower WAZ and HAZ throughout the study period.

Nutritional recovery was more common in the MUAC only cohort compared with the other cohorts, although the difference was not statistically significant in the MUAC only compared with WHZ only cohort (Table S2). In adjusted models, the MUAC and WHZ cohort had 0.27 times the odds of recovery compared with the MUAC only cohort (adjusted odds ratio [aOR] 0.27, 95% CI: 0.14–0.51). Three deaths were reported during the study, all of which were in the low MUAC and WHZ admission groups. Children in the low MUAC and WHZ group more often experienced fever compared with the MUAC‐only group (aOR 2.07, 95% CI: 1.24–3.45), but there was no significant difference between cohorts in malaria positivity by RDT or diarrhoea.

Children who were underweight or concurrently wasted and stunted at baseline, in general, had worse outcomes compared with children who were not underweight or concurrently wasted and stunted, respectively (Table S3). Among underweight children, 25% recovered compared with 44% of non‐underweight children (aOR 0.33, 95% CI: 0.19–0.58). Among concurrently wasted and stunted children, 14% recovered compared with 36% of those who were not concurrently wasted and stunted (aOR 0.22, 95% CI: 0.10–0.48). Underweight and concurrently wasted and stunted children gained weight faster than those who were not underweight or wasted and stunted (Table S3 and Figures S3 and S4). Underweight and concurrently wasted and stunted children had persistently lower MUAC, WHZ, WAZ and HAZ at 8 weeks compared with those not underweight or concurrently wasted and stunted, respectively (Figures S5 and S6).

## 4 DISCUSSION

In this analysis of children attending an outpatient SAM programme in Burkina Faso, children admitted to the programme based on both MUAC and WHZ had worse anthropometric indicators at admission and worse outcomes, including reduced odds of nutritional recovery and increased risk of signs of infection such as fever, compared with those admitted based only on a single criterion. MUAC alone has been proposed as a screening tool for acute malnutrition given its ease of use and based on evidence that low MUAC is a better predictor of mortality than low WHZ in community‐based settings (Ali et al., 2013; Briend et al., 2012, 2016; Chiabi et al., 2017; Gao et al., 2020; Goossens et al., 2012). Among those admitted to nutritional programmes, MUAC has been shown to identify children at the highest risk of mortality in some settings, although excluding those who met only WHZ criteria would have missed a substantial proportion of children who eventually died (Grellety et al., 2015). Other studies have demonstrated the importance of WHZ for identifying high‐risk children with SAM, and have suggested that children meeting both WHZ and MUAC criteria have the highest risk of mortality (Grellety and Golden, 2018). However, clinical and biochemical markers of risk of mortality and morbidity have been shown to be elevated in children admitted to SAM programmes based on WHZ or WHZ and MUAC compared with MUAC only (Dailey‐Chwalibóg et al., 2021). Although there were only three deaths in the current study, all were in children who met both WHZ‐ and MUAC‐based criteria, and anthropometric outcomes were worse in this group compared with MUAC‐ or WHZ‐only, supporting findings from previous studies.

Those enrolled based on low MUAC only had a more rapid rate of change in MUAC compared with those enrolled based on WHZ or both MUAC and WHZ. The trajectory of improvement in *Z*‐score‐based measures did not differ by admissions criteria, but deficits remained persistent over time for children admitted based on both WHZ and MUAC. This suggests a greater anthropometric deficit at baseline may contribute to worse outcomes at 8 weeks, even if change over time is consistent across categories. Children meeting both MUAC and WHZ criteria were less likely to achieve nutritional recovery than those admitted based solely on MUAC or WHZ, reflecting that they were at a worse starting point, and it may take more time and/or intensive intervention for them to achieve recovery. WHZ may provide additional information about which children have worse malnutrition above and beyond MUAC. In this study, WAZ < −3 identified nearly all of the low MUAC and low WHZ groups, the children who had the worst outcomes. Measurement of WAZ does not require height measurements and thus may be a more practical indicator for screening programmes and overcome some limitations of WHZ.

Previous studies have suggested that MUAC‐based screening identifies a different subset of children than WHZ (Laillou et al., 2014). For example, MUAC more often identifies younger female children compared with WHZ‐based screening (Berkley et al., 2005; Grellety et al., 2015). Similar to previous studies, children identified based solely on MUAC in the present study were more often female and on average were younger than those who met the WHZ criteria for SAM. MUAC increases as children age, which may partially explain why children were generally younger when identified with MUAC. WHZ has been shown to be more influenced by body shape (e.g., leg length) than MUAC (Myatt et al., 2009), which may lead to differences in estimations of the burden of acute malnutrition with each measure. In this study, a MUAC cutoff of 12.5 cm would have identified nearly all children with low WHZ. This cutoff is currently used to identify children with moderate acute malnutrition and may lead to an increase in false positives for SAM. Because this cohort only included children with SAM, we were unable to calculate the specificity of using a 12.5 cm cutoff for identifying children with WHZ < −3.

In addition to existing criteria used to admit children to the nutritional programme, we evaluated outcomes in subsets of children defined by WAZ (underweight) and WHZ and HAZ (concurrent wasting and stunting). WAZ has been shown to be a strong predictor of mortality in community‐based settings, and studies are increasingly recognizing the double burden of concurrent wasting and stunting that may place children at additional risk of poor outcomes (Gao et al., 2020; Mertens et al., 2020). Children with SAM who also met the criteria for underweight and concurrent wasting and stunting in this analysis, in general, had worse outcomes, including persistently lower *Z*‐scores for all measures across time points. Although WAZ and HAZ do not have the benefits of MUAC for ease of screening in community‐based settings, WAZ is easier to measure than WHZ or HAZ because it does not require a height measurement. Theoretically, children could be measured in the field relatively easily and WAZ could be calculated based on the weight measure and the child's age (Sié, Dah, et al., 2021; Sié, Ouattara et al., 2021). Nearly all children in this cohort had WAZ < −2; however, because all children had SAM, it is unclear how many children who would not meet the current SAM criteria would be admitted to the nutritional programme using that WAZ cutoff. A higher cutoff may unnecessarily overburden nutritional programmes with limited resources by admitting children who do not need as intensive treatment as those meeting other criteria.

This study must be considered in the context of several limitations. Data arose from a randomized controlled trial of azithromycin versus amoxicillin for SAM. All measurements were standardized and taken by trained anthropometrists as part of the trial, but the sample size was limited by the trial's overall objective (O'Brien, Sié, Dah, Ourohire, Arzika, et al., 2021; O'Brien, Sie, Dah, Ourohire, Ouédraogo, et al., 2021). CIs were wide for some comparisons, especially dichotomous outcomes, indicating uncertainty in those estimates. The duration of follow‐up was only 8 weeks, and thus we are unable to comment on longer‐term endpoints. Although a smaller proportion of participants enrolled based on both WHZ and MUAC recovered at 8 weeks compared with MUAC or WHZ only, it is possible that these proportions would have been more similar over time. Whether children admitted based on both WHZ and MUAC have persistently lower anthropometric measurements over the longer term or if they are more likely to relapse to SAM after recovery are important questions for understanding longer term consequences of any changes to screening criteria for SAM. Future follow‐up could include longer term time points as well as other clinical outcomes such as child development, body composition and long‐term health consequences. This study enrolled children attending outpatient nutritional programmes based out of primary health care facilities rather than dedicated nutritional centres. These health care facilities may have had fewer resources than one designed specifically for the management of SAM, which may limit generalizability in terms of how quickly children recovered, the proportion recovering, or the distribution of enrolment admissions criteria. Generalizability may also be limited by only including children who were admitted to a nutritional programme, and these results may not be generalizable to undernourished children who were not in treatment or to population‐based samples of children. In addition, the trial focused only on children with uncomplicated SAM who had not recently been admitted to a nutritional programme before the current SAM episode. The trial's inclusion criteria excluded children who likely had worse outcomes (e.g., those who have recently defaulted, those with complicated SAM), and this study's results can only be generalized to children meeting the trial's entry criteria. The overall recovery rate was lower than has been noted in some studies. While the reasons for this are unclear, it could be due to the 8‐week study time point or that enrolment facilities have fewer resources than facilities enrolling in previous studies. Finally, children were enrolled exclusively during the rainy season, which is the period of highest food insecurity in the Sahel. This may limit generalizability to children experiencing SAM during other times of the year, as the aetiology of SAM and profile of these cases may be different in different seasons.

In this secondary analysis of data arising from a randomized controlled trial of antibiotics for SAM, we found that children admitted based on both low MUAC and WHZ had worse anthropometric and clinical outcomes compared with children admitted based on a single criterion. MUAC‐only screening would have identified 86% of children ultimately enrolled in the trial. This analysis suggests that children admitted based on both MUAC and WHZ do not catch up to those meeting only one admission criterion in terms of anthropometric outcomes. Although MUAC is a much simpler screening tool than WHZ, those screening positive for SAM based on WHZ only had persistent anthropometric deficits and likely benefit from admission to the nutritional programme. Other indicators such as WAZ, which is easier to measure than WHZ as it does not require height measurements, may be considered to identify children at particularly high risk of poor outcomes.

## CONFLICT OF INTERESTS

None of the authors have any conflicts of interest to report.

## AUTHOR CONTRIBUTIONS

Clarisse Dah, Millogo Ourohire, Moussa Ouédraogo, Ali Sié, Moussa Ouédraogo, Mamadou Bountogo, Elodie Lebas, Benjamin F. Arnold, Kieran S. O'Brien and Catherine E. Oldenburg designed the study. Millogo Ourohire, Moussa Ouédraogo, Valentin Boudo, Elodie Lebas and Fanice Nyatigo supervised data collection. Valentin Boudo and Fanice Nyatigo developed the database and managed data cleaning. Fanice Nyatigo, Benjamin F. Arnold, Kieran S. O'Brien and Catherine E. Oldenburg analysed the data. Clarisse Dah and Catherine E. Oldenburg wrote the paper. All authors have read and approved the final manuscript.

## Supporting information

Supporting information.

## Data Availability

Data underlying these analyses are available from the corresponding author upon reasonable request.
