# Supplementary material for: How does baseline anthropometry affect anthropometric outcomes in children receiving treatment for severe acute malnutrition? A secondary analysis of a randomized controlled trial
Source: Matern Child Nutr. 2022 Feb 14;18(3):e13329. doi: 10.1111/mcn.13329 (PMC9218313; doi:10.1111/mcn.13329)
Supplement: Supplementary file 1 — Supporting information. [file MCN-18-e13329-s001.docx]

**Supplemental data for:** “Anthropometric outcomes following admission to outpatient treatment for uncomplicated severe acute malnutrition in children aged 6-59 months based on mid upper arm circumference and/or weight-for-height Z-score: a secondary analysis of a randomized controlled trial”

**Supplemental Table 1.** Baseline characteristics among children who were underweight (weight-for-age Z-score <-3) and concurrently wasted and stunted (weight-for-height Z-score <-3 and height-for-age Z-score <-3) at baseline

|  | **Underweight**  **(WAZ < -3)** | **Concurrently Wasted and Stunted**  **(WHZ < -3 and HAZ < -3)** |
| --- | --- | --- |
| N | 210 | 67 |
| Child’s age, months, median (IQR) | 15 (10 to 24) | 18 (13 to 23) |
| Female sex, N (%) | 109 (52%) | 22 (33%) |
| Child’s dietary diversity^1^, median (IQR) | 6 (4 to 7) | 5 (4 to 7) |
| Breastfeeding, N (%) | 144 (68%) | 48 (72%) |
| Household sanitation |  |  |
| None/bush | 30 (14%) | 9 (13%) |
| Unimproved latrine | 49 (23%) | 13 (19%) |
| Improved latrine | 110 (52%) | 36 (54%) |
| Flush toilet | 22 (10%) | 9 (13%) |
| MUAC, cm, mean (SD) | 11.2 (0.5) | 11.0 (0.7) |
| WHZ, mean (SD) | -3.4 (0.9) | -3.9 (0.6) |
| WAZ, mean (SD) | -4.0 (0.7) | -4.7 (0.5) |
| HAZ, mean (SD) | -3.0 (1.3) | -4.0 (0.8) |
| Mother’s age, years, median (IQR) | 25 (21 to 30) | 25 (22 to 30) |
| Mother is literate | 30 (14%) | 5 (7%) |

Abbreviations: MUAC, mid-upper arm circumference; WHZ, weight-for-height Z-score; WAZ, weight-for-age Z-score; HAZ, height-for-age Z-score; IQR, interquartile range; SD, standard deviation

^1^Composite variable assessing whether the child ate foods from the following 11 food groups in the last 7 days: grains, orange vegetables, green leafy vegetables, mangoes, other fruits, other vegetables, animal protein, eggs, nuts, dairy, or fat.

**Supplemental Table 2.** Probability of nutritional recovery, mortality, and morbidity events by 8 weeks by admission criteria

|  | N with outcome (%) | Odds Ratio  (95% CI) | Adjusted Odds Ratio^1^  (95% CI) |
| --- | --- | --- | --- |
| ***Nutritional recovery*** |  |  |  |
| MUAC only | 49 (50%) | 1.00 | 1.00 |
| WHZ only | 13 (34%) | 0.52 (0.24 to 1.13) | 0.38 (0.13 to 1.07) |
| WHZ and MUAC | 26 (17%) | 0.21 (0.12 to 0.37) | 0.27 (0.14 to 0.51) |
| ***Mortality*** |  |  |  |
| MUAC only | 0 (0%) | N/A | N/A |
| WHZ only | 0 (0%) |  |  |
| WHZ and MUAC | 3 (2%) |  |  |
| ***Malaria*** |  |  |  |
| MUAC only | 7 (7%) | 1.00 | 1.00 |
| WHZ only | 4 (11%) | 1.54 (0.42 to 5.61) | 1.47 (0.40 to 5.46) |
| WHZ and MUAC | 17 (11%) | 1.64 (0.65 to 4.11) | 1.64 (0.65 to 4.13) |
| ***Diarrhea^2^*** |  |  |  |
| MUAC only | 24 (24%) | 1.00 | 1.00 |
| WHZ only | 11 (27%) | 1.16 (0.51 to 2.66) | 1.30 (0.57 to 3.02) |
| WHZ and MUAC | 55 (34%) | 1.66 (0.94 to 2.91) | 1.69 (0.96 to 2.97) |
| ***Fever^3^*** |  |  |  |
| MUAC only | 43 (43%) | 1.00 | 1.00 |
| WHZ only | 21 (51%) | 1.39 (0.67 to 2.89) | 1.47 (0.70 to 3.11) |
| WHZ and MUAC | 97 (61%) | 2.04 (1.23 to 3.39) | 2.07 (1.24 to 3.45) |

Abbreviations: MUAC, mid-upper arm circumference; WHZ, weight-for-height Z-score.

^1^Adjusted for child’s age, sex (nutritional recovery, malaria, diarrhea, and fever) and baseline MUAC and baseline WHZ (nutritional recovery model only); ^2^Any experience of diarrhea reported during the study period; ^3^Any experience of fever reported during the study period

**Supplemental Table 3.** Outcomes by underweight (weight-for-age Z-score <-3) and concurrent wasting and stunting (weight-for-height Z-score <-3 and height-for-age Z-score <-3)

|  | **N with outcome (%) or Mean (SD)** | **Odds Ratio or Difference**  **(95% CI)** | **Adjusted Odds Ratio or Difference^1^**  **(95% CI)** |
| --- | --- | --- | --- |
| ***Nutritional recovery*** |  |  |  |
| Not underweight | 40 (44%) | 1.00 | 1.00 |
| Underweight | 49 (25%) | 0.41 (0.24 to 0.70) | 0.33 (0.19 to 0.58) |
| Not concurrently WaST | 80 (36%) | 1.00 | 1.00 |
| Concurrently WaST | 9 (14%) | 0.29 (0.13 to 0.61) | 0.22 (0.10 to 0.48) |
| ***Mortality*** |  |  |  |
| Not underweight | 0 (0%) | N/A | N/A |
| Underweight | 3 (1.5%) |  |  |
| Not concurrently WaST | 3 (1%) | N/A | N/A |
| Concurrently WaST | 0 (0%) |  |  |
| ***Malaria*** |  |  |  |
| Not underweight | 5 (6%) | 1.00 | 1.00 |
| Underweight | 23 (12%) | 2.26 (0.83 to 6.15) | 2.18 (0.77 to 6.06) |
| Not concurrently WaST | 17 (8%) | 1.00 | 1.00 |
| Concurrently WaST | 11 (17%) | 2.53 (1.12 to 5.72) | 2.60 (1.09 to 6.18) |
| ***Diarrhea^2^*** |  |  |  |
| Not underweight | 17 (19%) | 1.00 | 1.00 |
| Underweight | 73 (35%) | 2.30 (1.27 to 4.19) | 2.54 (1.38 to 4.70) |
| Not concurrently WaST | 67 (29%) | 1.00 | 1.00 |
| Concurrently WaST | 23 (34%) | 1.27 (0.72 to 2.27) | 1.41 (0.77 to 2.60) |
| ***Fever^3^*** |  |  |  |
| Not underweight | 48 (53%) | 1.00 | 1.00 |
| Underweight | 114 (54%) | 1.05 (0.64 to 1.72) | 1.01 (0.61 to 1.68) |
| Not concurrently WaST | 116 (50%) | 1.00 | 1.00 |
| Concurrently WaST | 46 (68%) | 2.13 (1.20 to 3.76) | 2.26 (1.25 to 4.09) |
| ***Weight gain (g/kg/day)*** |  |  |  |
| Not underweight | 1.7 (1.4) | Ref | Ref |
| Underweight | 3.0 (1.9) | 1.2 (0.8 to 1.7) | 1.4 (0.9 to 1.8) |
| Not concurrently WaST | 2.5 (1.7) | Ref | Ref |
| Concurrently WaST | 3.0 (2.2) | 0.5 (0.02 to 1) | 0.7 (0.2 to 1.3) |
| ***MUAC (cm)*** |  |  |  |
| Not underweight | 12.6 (0.8) | Ref | Ref |
| Underweight | 12.1 (0.8) | -0.5 (-0.7 to -0.3) | -0.5 (-0.7 to -0.3) |
| Not concurrently WaST | 12.4 (0.8) | Ref | Ref |
| Concurrently WaST | 11.9 (0.7) | -0.5 (-0.7 to -0.3) | -0.5 (-0.8 to -0.3) |
| ***WHZ*** |  |  |  |
| Not underweight | -1.7 (1.0) | Ref | Ref |
| Underweight | -2.0 (1.2) | -0.3 (-0.6 to -0.04) | 0.3 (0.04 to 0.6) |
| Not concurrently WaST | -1.7 (1.0) | Ref | Ref |
| Concurrently WaST | -2.6 (1.2) | -0.9 (-1.2 to -0.6) | -0.3 (-0.6 to 0.03) |
| ***WAZ*** |  |  |  |
| Not underweight | -1.8 (0.8) | Ref | Ref |
| Underweight | -3.0 (1.0) | -1.1 (-1.4 to -0.9) | 0.4 (0.1 to 0.7) |
| Not concurrently WaST | -2.3 (0.9) | Ref | Ref |
| Concurrently WaST | -3.7 (1.0) | -1.4 (-1.7 to -1.2) | -0.3 (-0.6 to -0.02) |
| ***HAZ*** |  |  |  |
| Not underweight | -1.2 (1.2) | Ref | Ref |
| Underweight | -2.9 (1.2) | -1.7 (-2.0 to -1.4) | -0.3 (-0.6 to -0.08) |
| Not concurrently WaST | -2.0 (1.4) | Ref | Ref |
| Concurrently WaST | -3.6 (1.0) | -1.7 (-2.1 to -1.3) | -0.2 (-0.5 to 0.04) |

Abbreviations: SD, standard deviation; WaST, concurrently wasted and stunted; MUAC, mid-upper arm circumference; WHZ, weight-for-height Z-score; WAZ, weight-for-age Z-score; HAZ, height-for-age Z-score; ^1^Adjusted for child’s age, sex (nutritional recovery, malaria, diarrhea, and fever) and baseline MUAC and baseline WHZ (nutritional recovery model only), and for baseline measure for MUAC, WHZ, WAZ, and HAZ outcomes; ^2^Any experience of diarrhea reported during the study period; ^3^Any experience of fever reported during the study period.

**Supplemental Figure 1.** Flow diagram of recruited participants based on admission criteria

**Excluded**

N=11 children

**Reasons for Exclusion**

- Recent antibiotic use: 3
- Inpatient treatment: 1
- Recent admission to nutritional program: 6
- Residence outside catchment area: 2
- Not available for full study period: 1

**Refused**

N=0 children

**Assessed for eligibility**

N=312 children

**Enrolled**

N=301 children

**MUAC Only**

N=100 children

**MUAC and WHZ**

N=160 children

**WHZ Only**

N=41 children

**Recovered:** 49

**Not recovered:** 49

**Died:** 0

**Lost to follow-up:** 2

**Recovered:** 26

**Not recovered:** 124

**Died:** 3

**Lost to follow-up:** 7

**Recovered:** 13

**Not recovered:** 25

**Died:** 0

**Lost to follow-up:** 3

**Supplemental Figure 2.** Venn diagram depicting number of children enrolled in categories defined by criteria used for admission to the outpatient nutritional program (weight-for-height Z-score [WHZ] < -3, green, and/or mid-upper arm circumference [MUAC] < 11.5 cm, brown) and weight-for-height Z-score (WAZ) < -2 (blue) and height-for-age Z-score (HAZ) < -2 (yellow) at enrollment.

**
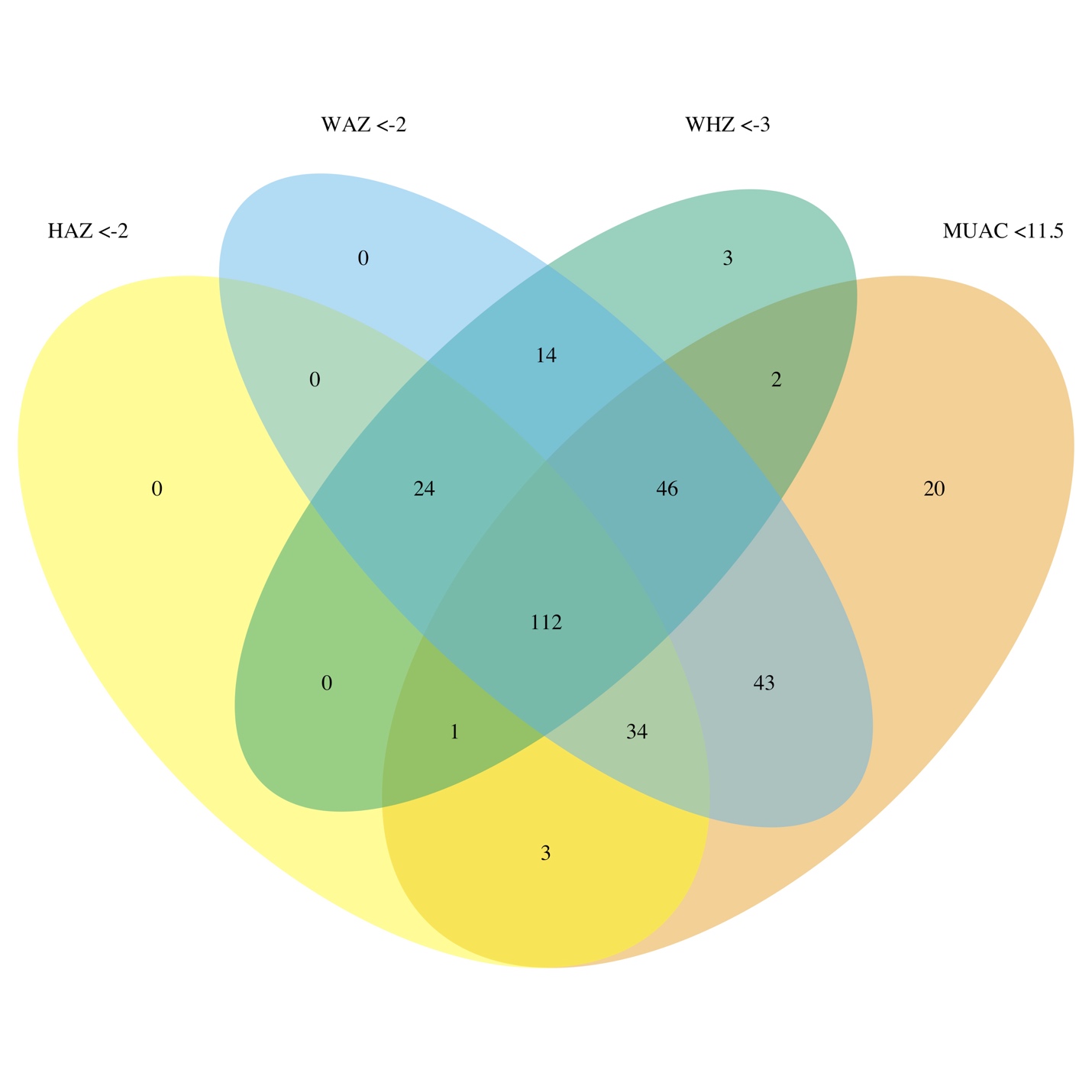
**

**Supplemental Figure 3.** Weight gain in g/kg/day over time among children who were underweight (weight-for-age Z-score < -3) at enrollment compared to not underweight at enrollment. Underweight children are in black and not underweight are in grey. Points represent mean growth between the two study time points indicated on the x axis and bars represent 95% confidence intervals.

**Supplemental Figure 4.** Weight gain in g/kg/day over time among children who had both low weight-for-height Z-score (WHZ) and height-for-age Z-score (HAZ, as defined by WHZ < -3 and HAZ < -3) at enrollment compared to not concurrently stunted and wasted at enrollment. Concurrently stunted and wasted children are in black and not are in grey. Points represent mean growth between the two study time points indicated on the x axis and bars represent 95% confidence intervals.

**Supplemental Figure 5.** Mid-upper arm circumference (MUAC; A), weight-for-height Z-score (WHZ; B), weight-for-age Z-score (WAZ; C), and height-for-age Z-score (HAZ; D) over time by underweight (weight-for-age Z-score < -3) at enrollment. Underweight children are in black and those not underweight are in grey. Points represent means for the study time point and bars represent 95% confidence intervals.

**Supplemental Figure 6.** Mid-upper arm circumference (MUAC; A), weight-for-height Z-score (WHZ; B), weight-for-age Z-score (WAZ; C), and height-for-age Z-score (HAZ; D) over time by concurrent wasting and stunting (weight-for-height Z-score and height-for-age Z-score < -3) at enrollment. Concurrently wasted and stunted children are in black and those not are in grey. Points represent means for the study time point and bars represent 95% confidence intervals.
